# Machine Learning-Based Identification of Hub Genes and Temporal Regulation Mechanisms in Zebrafish Fin Regeneration

**DOI:** 10.3390/genes17050503

**Published:** 2026-04-24

**Authors:** Xiaoying Jiang, Junli Zheng, Yuqin Shu, Yinjun Jiang, Cheng Guo

**Affiliations:** 1 State Key Laboratory of Developmental Biology of Freshwater Fish, Engineering Research Center of Polyploid Fish Reproduction and Breeding of the State Education Ministry, College of Life Sciences, Hunan Normal University, Changsha 410081, China; 2 Department of Clinical Laboratory, The Quzhou Affiliated Hospital of Wenzhou Medical University, Quzhou People’s Hospital, Quzhou 324000, China

**Keywords:** zebrafish (*Danio rerio*), fin regeneration, machine learning, WGCNA, TO-GCN

## Abstract

**Background/Objectives**: Zebrafish fin regeneration serves as a classic model for investigating vertebrate tissue regeneration, yet the core regulatory networks and their crosstalk with the immune microenvironment remain incompletely characterized. This study aimed to identify hub genes, and elucidate the underlying molecular mechanisms and immune microenvironment dynamics during zebrafish fin regeneration. **Methods**: We integrated multiple bulk RNA-seq datasets of zebrafish fin regeneration from the GEO database, followed by data standardization with batch effect removal. Hub genes were screened via differential expression analysis, weighted gene co-expression network analysis (WGCNA), and predictive models constructed with 13 classic machine learning algorithms. Functional enrichment, time-ordered gene co-expression network (TO-GCN) method, immune infiltration analyses and RT-qPCR validation were further performed. **Results**: We identified upregulated differentially expressed genes, regeneration-correlated gene modules and their overlapping genes, including 82 candidate genes and 10 hub genes enriched in cytoskeleton remodeling, extracellular matrix organization, and focal adhesion. Temporal analysis uncovered hierarchical gene regulation and functional switching during regeneration. Hub gene expression was significantly correlated with the infiltration of B cells, M1/M2 macrophages and CD8+ T cells, revealing a stage-specific immune microenvironment. RT-qPCR validation showed high consistency with the multi-omics data. **Conclusions**: This study provides potential gene targets for understanding zebrafish fin regeneration, and offers a valuable reference for investigating the crosstalk between regulatory networks and the immune microenvironment in vertebrate tissue regeneration.

## 1. Introduction

Regenerative biology is a core research field with significant fundamental research value and practical implications for animal science. Its key scientific question lies in deciphering the intrinsic molecular regulatory mechanisms underlying the structural reconstruction and functional recovery of damaged tissues or organs in organisms, thereby providing theoretical basis and model references for addressing critical issues in animal biology such as aquatic vertebrate tissue repair, farmed fish health, and animal welfare. Significant interspecies differences exist in the regenerative capacity of vertebrates. As a typical lower vertebrate, zebrafish (*Danio rerio*) possesses robust regenerative capacity for tissues and organs including the heart, spinal cord, and retina [1,2,3,4,5]. After injury, its caudal fin can achieve precise morphological and functional reconstruction within weeks through ordered cell proliferation, differentiation, migration, and tissue remodeling [6]. Moreover, the molecular regulatory network of zebrafish fin regeneration is highly conserved with that of other aquatic vertebrates, making it an ideal model organism for deciphering the mechanisms of vertebrate appendage regeneration and providing direct reference for tissue repair in farmed fish [7,8,9]. Zebrafish fin regeneration is a highly complex and dynamically regulated biological process, involving the coordination of multiple levels such as spatiotemporal specific changes in gene expression, intercellular signal communication, and stage-specific regulation of the immune microenvironment [10,11,12]. Comprehensive identification of the responsive genes and regulatory networks in this process is a crucial breakthrough for understanding vertebrate regeneration mechanisms.

With the rapid development of high-throughput sequencing technology and the continuous improvement of public data platforms, a large number of transcriptomic datasets related to zebrafish fin regeneration have been accumulated and shared, providing abundant data support for deciphering the molecular basis of regeneration at the whole-genome level [13,14,15]. Currently, existing studies have revealed partial molecular regulatory characteristics of zebrafish fin regeneration using RNA-seq technology [16,17]. For instance, Lee et al. found that the epigenome of regenerating zebrafish fins exhibits stable maintenance of lineage-specific DNA methylation and dynamic changes in chromatin accessibility, and the open state of chromatin provides an epigenetic basis for the transcriptional activation of regeneration-related genes [18]; Thompson et al. identified a set of specific enhancers that regulate the spatiotemporal expression of genes during zebrafish fin regeneration, confirming the key role of cis-regulatory elements in regeneration [19]; Li et al. discovered that heat shock protein Hsp90α, cytokine family genes, and long non-coding RNA lincRNA-154324, together with its adjacent protein-coding gene *vmp1*, are involved in the regulation of zebrafish caudal fin regeneration, revealing the synergistic role of coding genes and non-coding RNAs in regeneration [20,21]. These studies have uncovered partial molecular characteristics of zebrafish fin regeneration from perspectives such as epigenetics, transcriptional regulation, and non-coding RNA regulation. However, limited by research methods and data scale, numerous issues remain to be addressed. Meanwhile, most existing studies adopt a single bioinformatics analysis method, either focusing solely on differentially expressed genes or analyzing the function of individual genes, failing to construct the molecular regulatory network of regeneration from the perspective of gene co-expression patterns and core feature screening, which hinders the accurate identification of key responsive genes during regeneration. In addition, as a dynamic temporal process, zebrafish fin regeneration exhibits significant stage-specific gene expression [22,23], but existing studies lack in-depth analysis of the hierarchical gene regulatory network at different regeneration stages. Furthermore, the immune microenvironment, as an important regulatory factor in the regeneration process, its interaction mechanism with core genes has not been systematically revealed. These research gaps have become major obstacles to a deeper understanding of the molecular mechanisms of zebrafish fin regeneration.

Integrative analysis of multi-source public transcriptomic datasets is an effective approach to overcome the limitations of single datasets and decipher complex biological processes at the whole-genome level. It can improve the statistical power of research by expanding the sample size and more comprehensively capture the molecular characteristics of biological processes. However, differences in experimental platforms, sample processing conditions, and detection batches among different datasets can lead to significant batch effects, resulting in non-biological biases in the data. Direct integrative analysis without correction will seriously affect the reliability of results. Therefore, efficient batch effect correction is a prerequisite and key for multi-source data integration [24]. Currently, the ComBat method [25] based on empirical Bayes is a classic approach for batch effect correction of transcriptomic data, which can effectively eliminate non-biological biases caused by batches while retaining biological differences, and has been widely applied in the integrative analysis of multi-source high-throughput data [26]. Traditional differential expression analysis (DE analysis) can identify changes in gene expression under different phenotypes, but it only reflects the expression characteristics of individual genes and cannot reveal the co-expression patterns among genes. As a systems biology method, weighted gene co-expression network analysis (WGCNA) can construct co-expression networks based on gene expression correlations, identify gene modules highly associated with phenotypes, and reflect the synergistic regulatory relationships among genes [27]. Combining DE analysis with WGCNA can narrow down the range of candidate genes, but more precise feature screening methods are still required to identify core responsive genes.

Machine learning algorithms, with their powerful capabilities in feature extraction, pattern recognition, and prediction, have become important research tools in the field of bioinformatics, and are widely used in disease biomarker identification, core functional gene screening, biological phenotype prediction, and other studies [28,29,30,31]. By constructing multiple machine learning models and evaluating their performance, followed by selecting high-performance algorithms for core feature screening, the accuracy and reliability of key gene identification can be significantly improved. Currently, machine learning algorithms have been maturely applied in fields such as oncology [32] and neurobiology [30,33], but their application in zebrafish fin regeneration research is relatively limited. No studies have yet used the combined analysis of multiple machine learning algorithms to screen core hub genes of zebrafish fin regeneration at the whole-genome level, which provides an important entry point for this study.

In addition to the screening of core genes, deciphering the temporal expression patterns of genes and the regulatory role of the immune microenvironment is also crucial for a comprehensive understanding of the molecular mechanisms of zebrafish fin regeneration. Zebrafish fin regeneration can be divided into multiple stages including wound healing, blastema formation, proliferation and differentiation, and tissue remodeling. Gene expression characteristics vary significantly among different stages, forming a dynamic temporal regulatory network [9,34]. Traditional gene expression analysis methods are difficult to accurately construct temporal gene regulatory networks. The time-ordered gene co-expression network (TO-GCN) method integrates co-expression information from different time points and introduces temporal order to construct hierarchical regulatory networks, which can effectively reveal the temporal regulatory characteristics of genes in dynamic biological processes [35]. Meanwhile, the immune microenvironment plays a dual role in tissue regeneration: an appropriate inflammatory response can initiate the regeneration program, while the timely resolution of inflammation provides conditions for tissue repair and remodeling. There is a close interaction between the infiltration characteristics of immune cells and the expression of core genes [36,37]. Using immune infiltration analysis to decipher changes in the composition of immune cells during regeneration and exploring their correlation with core genes can reveal the synergistic mechanism between the immune microenvironment and the molecular regulatory network, and improve the understanding of the zebrafish fin regeneration process.

Based on the above research background and gaps, this study integrated three bulk RNA-seq datasets related to zebrafish fin regeneration from the Gene Expression Omnibus (GEO) public database (GSE126701, GSE146960, GSE160909). First, the ComBat method from the sva package was used for batch effect correction, and t-SNE dimensionality reduction clustering analysis was performed to verify the correction effect, achieving standardized integration of multi-source data. Subsequently, the Limma package was used for DE analysis, combined with WGCNA to construct a gene co-expression network, and screen core gene sets highly associated with the regeneration phenotype. On this basis, 13 classic machine learning algorithms were used to construct prediction models for the zebrafish fin regeneration phenotype. ROC curves and AUC values were used to evaluate model performance, and high-performance algorithms were selected to integrate their feature screening results to identify regeneration-related hub genes. Furthermore, KEGG and GO functional enrichment analyses were performed to decipher the biological functions of hub genes and the signaling pathways they participate in. The TO-GCN method was used to construct a temporal gene co-expression network, revealing the temporal regulatory characteristics of genes during regeneration. Finally, the immunedeconv package was used for dual immune infiltration analysis (ESTIMATE and quanTIseq) to explore the characteristics of immune cell infiltration during regeneration, and analyze the correlation between hub genes and differential immune cells, revealing the interaction mechanism between the immune microenvironment and core genes. This study systematically identifies regeneration-responsive genes during zebrafish fin regeneration, analyses their molecular regulatory networks, temporal expression characteristics, and immune microenvironment regulatory mechanisms, providing experimental evidence and theoretical support for a deeper understanding of the molecular basis of vertebrate appendage regeneration.

## 2. Materials and Methods

### 2.1. Data Collection and Batch Effect Removal

Bulk RNA-seq transcriptomic datasets related to the molecular mechanisms of zebrafish fin regeneration and corresponding sample phenotypic information were downloaded from the GEO public database at https://www.ncbi.nlm.nih.gov/geo/ (accessed on 2 December 2025). A total of three zebrafish fin regeneration-related datasets were included, namely GSE126701 [18], GSE146960 [19], and GSE160909 [20,21]. Raw datasets were subjected to preliminary sample screening to strictly exclude samples with missing phenotypic information or incomplete annotations. Ultimately, only samples directly related to zebrafish fin regeneration were retained for subsequent integrative analysis to ensure the targeting of research samples and the validity of data. For effective integration of multi-source datasets and elimination of non-biological biases caused by different experimental batches and detection platforms, the ComBat algorithm from the R package sva (v3.46.0) [24,25] was used to correct batch effects of the screened multi-source transcriptomic expression data based on an empirical Bayesian correction model. To verify the effectiveness of batch effect removal, t-distributed Stochastic Neighbor Embedding (t-SNE) dimensionality reduction clustering analysis was performed on the integrated datasets before and after correction using the R package Rtsne (v0.16) [38] to determine the elimination effect of batch effects. And silhouette coefficients were computed using batch labels and Euclidean distance with the cluster R package (v2.1.8.1) to quantitatively assess the effectiveness of batch effect removal.

### 2.2. Screening of Key Genes

DE analysis was performed on the batch effect-corrected integrated dataset using the Limma package (v3.62.2) [39]. Stabilization of variance estimation was achieved through a classic Bayesian method, and differentially expressed genes (DEGs) related to zebrafish fin regeneration were identified with |logFC| > 1 and adjusted *p*-value < 0.001 as screening thresholds.

WGCNA was used to decipher the co-expression patterns among genes. This method quantifies the strength of expression correlations between genes (nodes) and assigns corresponding weights, uses the Topological Overlap Matrix (TOM) to measure the degree of co-expression association between genes, and further identifies gene modules with highly synergistic expression patterns [27]. Using the R package WGCNA (v1.72-5) to sequentially complete optimal soft threshold screening, gene clustering, co-expression module identification, and association analysis between modules and phenotypic traits. Core co-expression modules significantly associated with the zebrafish fin regeneration phenotype were screened, and key module genes were extracted.

The DEGs screened above were intersected with the key module genes in the core co-expression modules to obtain a key gene set related to zebrafish fin regeneration, which served as the candidate gene set for subsequent machine learning model construction.

### 2.3. Construction and Evaluation of Machine Learning Models

The 13 machine learning algorithms were selected based on their complementary strengths in handling high-dimensional transcriptomic data, and implemented using various R packages with an 8:2 random split of the dataset into training and validation sets via an in-house Python script. Specifically, AdaBoost (adabag v5.0, 30 iterations) [28] and XGBoost (xgboost v1.7.5.1, learning rate 0.3, max depth 6) [29] were chosen for their robustness in capturing complex feature interactions; Lasso, Ridge, and Elastic Net (glmnet v4.1-7, Alpha = 0.5 for Elastic Net) [30] for regularization and feature selection; neural network (NeuralNetTools v1.5.3 and neuralnet v1.44.2, rprop+ training, hidden layer 30 × 30) [31] for modeling nonlinear relationships; PLS (pls v2.8-2, importance score threshold 0.015) [40] and SuperPC (superpc v1.12) [41] for dimensionality reduction; CatBoost (catboost v1.2, 30 iterations) [42] for efficient handling of categorical features; Random Forest (randomForest v4.7-1.1) [43] and Decision Tree (rpart v4.1.19) [44] for interpretability and resistance to overfitting; GLM (caTools v1.18.2) [45] as a baseline linear model; and SVM RFE (e1071 v1.7-13) [46] for recursive feature elimination.

To ensure robust performance evaluation and reduce the risk of overfitting, ten-fold cross-validation was applied during model training. Specifically, the training set was randomly partitioned into ten equal subsets. In each iteration, nine subsets were used for training and the remaining one for validation, and this process was repeated ten times. The final model performance was averaged across all ten folds. All hyperparameters were optimized within the cross-validation loop. This strategy provides a more reliable estimate of model generalizability than a single train-validation split.

To evaluate model performance, receiver operating characteristics (ROC) analysis was performed using the pROC package (v1.18.4) [47], and the constructed models and screened features were evaluated by calculating the area under the curve (AUC) values.

### 2.4. Functional Annotation and Enrichment Analysis

Functional annotation and enrichment analysis of Kyoto Encyclopedia of Genes and Genomes (KEGG) and Gene Ontology (GO) were implemented using the clusterProfiler package (v4.6.2) [48]. The former was used to identify significantly enriched signaling pathways, while the latter was used to evaluate the distribution of candidate genes in biological processes (BP), molecular functions (MF), and cellular components (CC).

### 2.5. Analysis of Gene Expression Patterns Under Time Series

TO-GCN analysis was used to decipher the temporal regulatory characteristics of genes during zebrafish fin regeneration, and the Breadth-First Search (BFS) algorithm was used to introduce temporal order features, ultimately constructing a gene regulatory hierarchical network capable of revealing the molecular mechanisms of dynamic biological processes [35]. Official annotation information of zebrafish transcription factors (TFs) was obtained from the Animal Transcription Factor Database (AnimalTFDB 4.0) [49]. A gene co-expression network (GCN) was constructed based on this annotation information and the integrated temporal transcriptomic data, and the regeneration-specific GCN (C1+C20) was ultimately selected to analyze the temporal co-expression regulatory relationships of hub genes.

The Mfuzz package (v2.66.0) was used for Fuzzy C-Means Clustering (FCM) analysis to identify temporal expression clusters of zebrafish fin regeneration-related genes. The optimal number of clusters was determined with the minimum cluster center distance as the criterion, and the expression pattern characteristics of genes in different clusters during the temporal process of fin regeneration were systematically analyzed based on the clustering results [50].

### 2.6. Immune Infiltration Analysis and Correlation Analysis Between Hub Genes and Immune Cells

The immunedeconv package (v2.1.0) was used to perform immune infiltration analysis on the integrated dataset using two deconvolution-based algorithms, ESTIMATE and quanTIseq [37], to decipher the immune microenvironment characteristics of zebrafish caudal fin regeneration. Immune cell types with significantly different infiltration levels were screened, and the correlation between these cell types and the expression levels of hub genes was calculated based on Spearman’s rank correlation coefficient.

### 2.7. Quantitative Real-Time PCR (qPCR) Validation

Wild-type AB strain zebrafish (4 months old, body length 3.5–4.0 cm) were obtained from the National Aquatic Biological Resource Center. Zebrafish were reared in a recirculating aquaculture system at 28 ± 0.5 °C with a 14 h light/10 h dark photoperiod and fed with commercial feed twice daily. All animal experiments were approved by the Animal Ethics Committee of Hunan Normal University. Zebrafish caudal fin amputation was performed as previously described with minor modifications. Briefly, zebrafish were anesthetized with 0.02% tricaine methanesulfonate (MS-222), and the caudal fin was amputated at 50% of its total length using sterile surgical scissors. After amputation, the fish were quickly transferred to fresh water for recovery. Caudal fin tissues were collected at 0, 3, 5, 7 and 10 days post-amputation (dpa) (*n* = 9 fish per time point). Tissues were immediately frozen in liquid nitrogen and stored at −80 °C until RNA extraction.

Total RNA was extracted from caudal fin tissues using TRIzol^®^ Reagent (Invitrogen, Carlsbad, CA, USA) following the manufacturer’s instructions. The purity and concentration of RNA were determined using a NanoDrop 2000 spectrophotometer (Thermo Fisher Scientific, Waltham, MA, USA). RNA integrity was verified by 1.2% agarose gel electrophoresis. Reverse transcription was performed by incubating 1 μg of RNA with 2.5 μM oligo dT primer, 1 mM deoxynucleotide triphosphate mixture, 20 U RNase inhibitor, and 100 U RevertTra Ace (Toyobo Life Science, Shanghai, China) in the appropriate buffer for 60 min at 42 °C and 5 min at 99 °C. Quantitative PCR was performed on a Bio-Rad CFX96 Real-Time PCR System (Bio-Rad, Hercules, CA, USA) using SYBR Green Realtime PCR Master Mix (Toyobo Life Science, Shanghai, China). The reaction conditions were 95 °C for 3 min, followed by 40 cycles of 95 °C for 15 s, 60 °C for 15 s, and 72 °C for 15 s [51]. The primers used for amplification are listed in Table 1. The expression values of the target genes were normalized to the amount of *β-actin* mRNA. Data analysis was performed using the 2^−ΔΔCT^ method [52]. Each experiment was performed in triplicate.

Statistical analysis was performed using SPSS 26.0 software (IBM, Armonk, NY, USA). Data are presented as the mean ± standard error of the mean (SEM). One-way analysis of variance (ANOVA) followed by Tukey’s post hoc test was used to compare expression differences among different time points. Differences were considered statistically significant at *p* < 0.05.

## 3. Results

### 3.1. Identification of Regeneration-Related Differentially Expressed Genes and Screening of Co-Expression Core Modules

**Figure 1:**
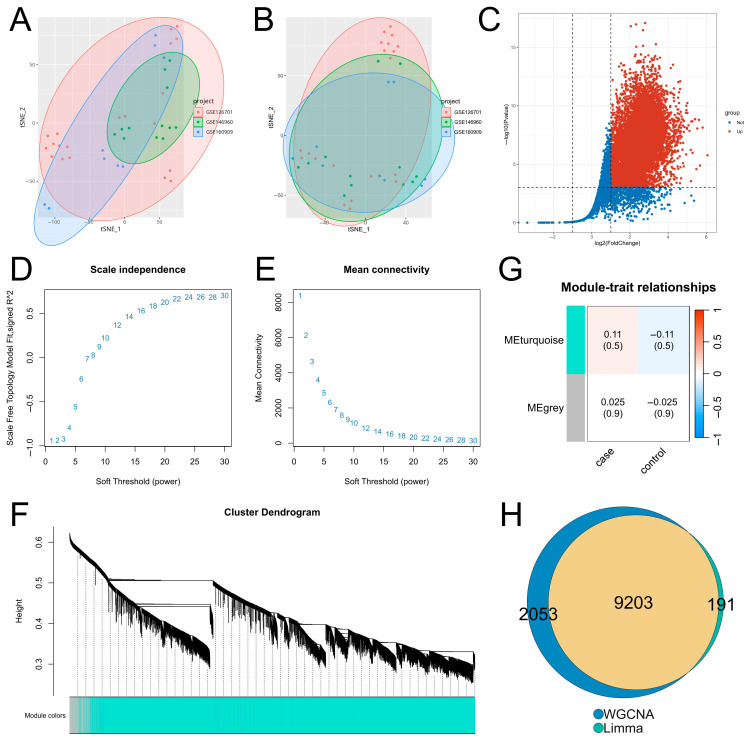
Results of DE analysis and WGCNA. (**A**,**B**) Results of t-SNE dimensionality reduction clustering analysis of the integrated dataset before and after batch effect removal. (**C**) Volcano plot of DE analysis results. (**D**,**E**) Selection of the optimal soft threshold. (**F**) Construction of gene co-expression modules. (**G**) Results of correlation analysis between gene modules and phenotypic traits. (**H**) Venn diagram of the intersection between DE analysis and WGCNA results.

After batch effect correction of multi-source zebrafish fin regeneration transcriptomic datasets, t-SNE dimensionality reduction clustering analysis was performed to verify the correction effect. As shown in the clustering results before and after correction (Figure 1A,B), batch effects were significantly eliminated. The mean silhouette score decreased from 0.0295 before correction to 0.0039 after ComBat correction, indicating effective elimination of batch effects (Supplementary Table S2).

DEGs related to zebrafish fin regeneration were identified with |logFC| > 1 and adjusted *p*-value < 0.001 as screening thresholds—the former is a gold standard for identifying biologically meaningful expression changes in transcriptomic research to exclude random low-amplitude fluctuations, and the latter is a strict FDR correction adapted to the expanded sample size of multi-dataset integration, which effectively minimizes false positive results [18,19]. In this study, we only focused on upregulated DEGs because fin regeneration is an active injury-induced biological process, and transcriptional activation of genes is the core molecular driver of regeneration program initiation and progression, while downregulated genes are mostly quiescence-maintaining genes with no direct regulatory role in regeneration [13,18,19,20]. DE analysis results revealed (Figure 1C, Supplementary Table S3) that 9394 genes were significantly upregulated during the regeneration process of zebrafish fins after injury.

To identify modules significantly associated with zebrafish fin regeneration, WGCNA was applied to the integrated dataset. As shown in Figure 1D,E, the power at the inflection point optimally balances the scale-free property of the network and the integrity of gene co-expression relationships. When the soft threshold power was set to 24, the signed R^2^ reached 0.86, and the average connectivity of all nodes was at a low level, indicating that the network conformed to the distribution of a scale-free network. Based on the optimal soft threshold (power = 24) and expression profile data, an adjacency matrix was calculated for gene clustering analysis. As shown in Figure 1F, genes clustered into the same branch were assigned to the same module. Association analysis between gene modules and phenotypic traits (Figure 1G, Supplementary Table S4) showed that only two modules (MEturquoise and MEgrey) were identified, with the MEturquoise module having the highest correlation with phenotypic traits, suggesting that the genes in this module may have functional significance related to zebrafish fin regeneration.

The regeneration-related upregulated differentially expressed genes identified above were intersected with the genes in the MEturquoise module. As shown in Figure 1H, a total of 9203 overlapping genes were obtained, which served as the basic gene set for subsequent screening of regeneration core genes.

### 3.2. Construction and Evaluation of Machine Learning Models for Screening Regeneration-Related Hub Genes

**Figure 2:**
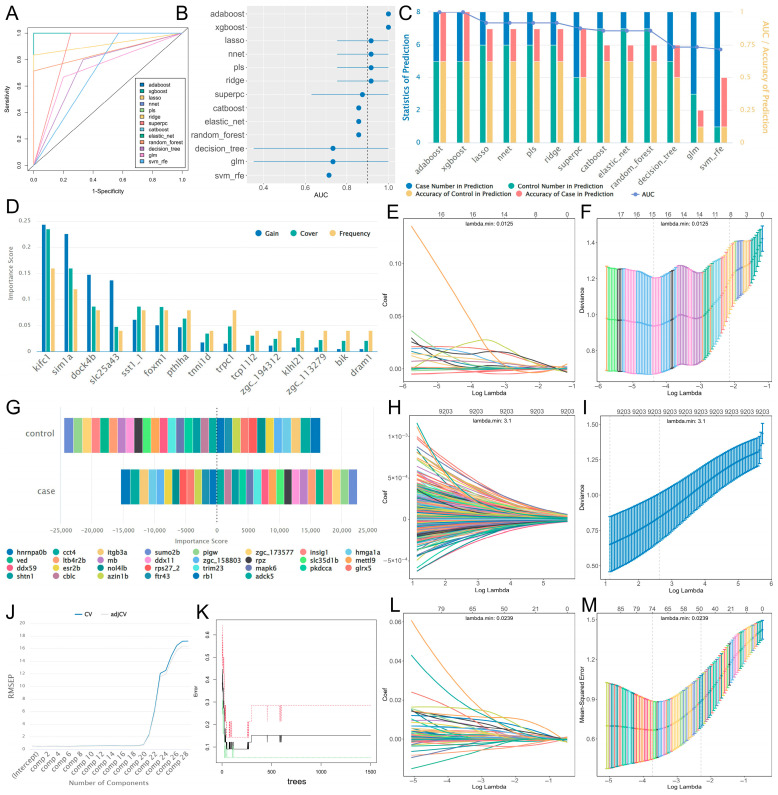
Results of analysis based on 13 classic machine learning algorithms. (**A**) ROC curves of all models. (**B**) Forest plot of AUC values of all models, the horizontal lines represent the 95% confidence intervals for the AUC values of each model (detailed values are provided in Supplementary Table S7). (**C**) Statistics of prediction results for the validation set. (**D**) Statistical bar chart of gain, cover, and frequency for XGBoost. (**E**,**F**) Regression coefficient path diagrams and cross-validation curves for Lasso. (**G**) Statistics of feature contribution for the neural network. (**H**,**I**) Regression coefficient path diagrams and cross-validation curves for Ridge. (**J**) Error curve of PLS. (**K**) Out-of-Bag (OOB) error trend plot of Random Forest. The black, red, and green lines indicate the overall OOB error, the OOB error for the regeneration group, and the OOB error for the control group. (**L**,**M**) Regression coefficient path diagrams and cross-validation curves for Elastic Network.

To identify hub genes related to zebrafish fin regeneration, feature engineering based on 13 classic machine learning algorithms was performed using the training set to construct prediction models, and the performance of the 13 algorithm models was evaluated using the validation set. ROC curves and AUC values (Figure 2A,B) showed that all 13 models exhibited good performance, with AUC values above 0.7. Among them, AdaBoost, XGBoost, Lasso, neural network, PLS, and Ridge performed the best, with AUC values reaching above 0.9. Statistical analysis of the prediction results of these models (Figure 2C) showed that the prediction accuracy of AdaBoost and XGBoost was as high as 100%, while Lasso, neural network, PLS, and Ridge had errors in the prediction of injured samples, but the overall accuracy still exceeded 85%, indicating that these models had high robustness and reliability.

Among them, the bar chart of XGBoost algorithm results (Figure 2D) showed three important indicators of core genes in the model, namely gain, cover, and frequency, which respectively measure the relative contribution of features to model accuracy, the relative number of observations involved in features, and the proportion of times features appear in model trees. The stacked bar chart of the neural network model presented the contribution degree of core genes to each category (Figure 2G). The error curve of PLS showed that when the number of principal components was 7, the root mean square error of prediction (RMSEP) of the model was the lowest (Figure 2J). The Out-of-Bag (OOB) error trend plot of the Random Forest algorithm indicated that when the number of decision trees was greater than 700, the internal error of the model tended to stabilize (Figure 2K). The regression coefficient path diagrams of Lasso, Ridge, and Elastic Net revealed that as the regularization parameter λ increased, the coefficients of each feature gradually converged, and the cross-validation curves indicated that the minimum λ values corresponding to the optimal models were 0.0125, 3.1, and 0.0239, respectively (Figure 2E,F, Figure 2H,I, Figure 2L,M).

### 3.3. Hub Gene Screening and Functional Study

**Figure 3:**
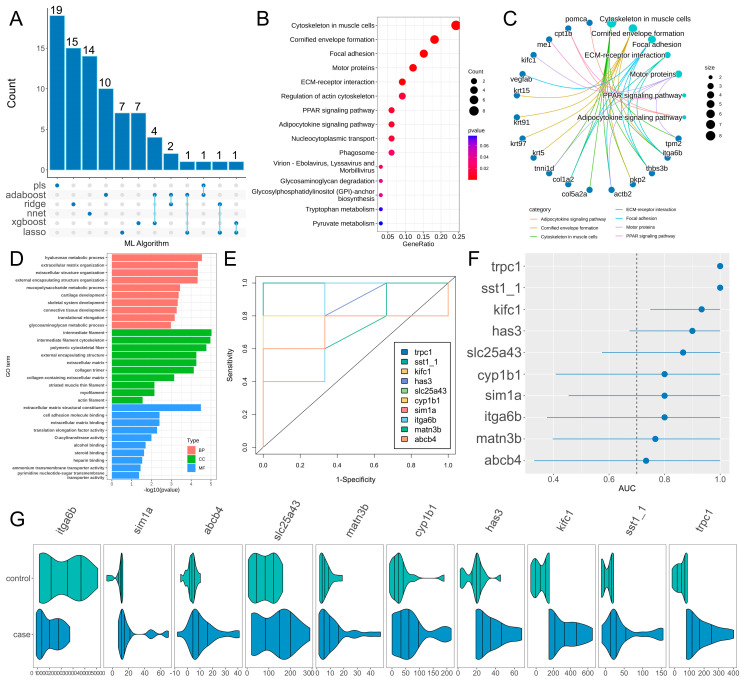
Results of hub gene screening and functional analysis. (**A**) Upset plot of feature screening results from the six top-performing models. (**B**,**C**) Results of KEGG enrichment analysis for the candidate gene set. (**D**) Results of GO enrichment analysis for the candidate gene set. (**E**) ROC curves of hub genes. (**F**) Forest plot of AUC values for hub genes. (**G**) Expression patterns of hub genes.

Generally speaking, a model with an AUC value exceeding 0.9 is typically considered an excellent classifier. The genes identified by the six high-performance algorithm models were integrated to obtain a candidate gene set containing 82 genes (the union of the top 30 genes from each model) (Figure 3A). Subsequently, KEGG and GO functional annotation and enrichment analysis were performed on the candidate gene set. KEGG enrichment results (Figure 3B,C, Supplementary Table S6) showed that the candidate genes were mainly enriched in pathways such as cytoskeleton in muscle cells, cornified envelope formation, focal adhesion, motor proteins, and ECM–receptor interaction. GO enrichment results (Figure 3D, Supplementary Table S5) showed that regarding biological process (BP), the genes were mainly enriched in terms of hyaluronan metabolic process, extracellular matrix organization, and extracellular structure organization; regarding cellular component (CC), they were mainly enriched in terms of as intermediate filament, intermediate filament cytoskeleton, and polymeric cytoskeletal fiber; regarding Molecular Function (MF), they were mainly enriched in terms of extracellular matrix structural constituent, cell adhesion molecule binding, and extracellular matrix binding.

Among the candidate gene set, 10 overlapping genes were identified by at least two algorithms as hub genes. ROC curve analysis of the hub genes (Figure 3E,F) showed that all hub genes exhibited excellent class discrimination ability. Among them, *trpc1*, *sst1.1*, *kifc1*, *has3*, *cyp1b1*, *matn3b*, *slc25a43*, *abcb4*, *sim1a* and *itga6b* were significantly upregulated in injured regenerating samples.

### 3.4. Analysis of Gene Expression Patterns and Functions Under Time Series

**Figure 4:**
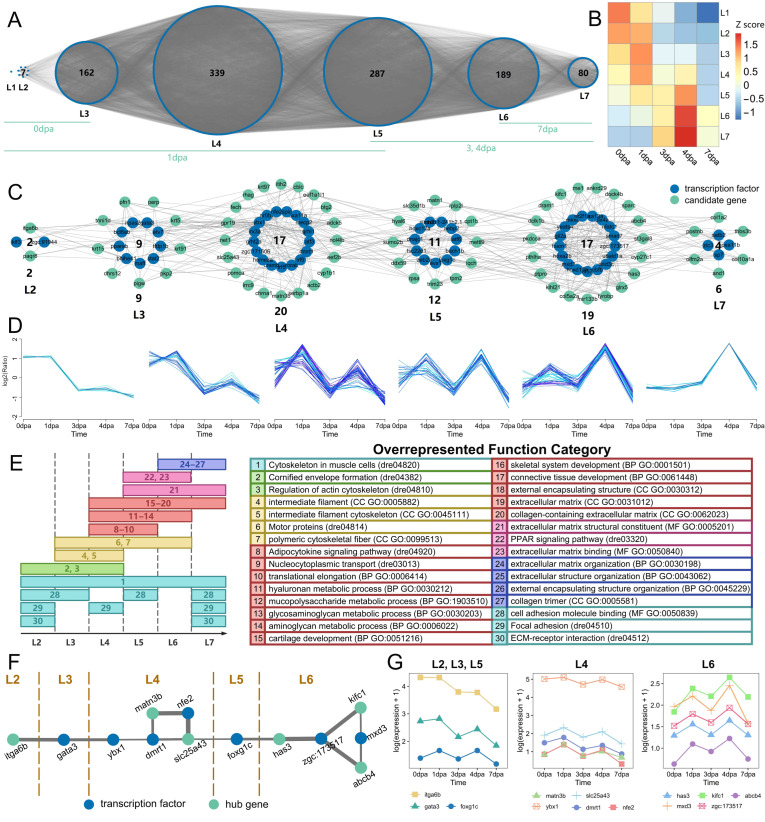
Gene expression and functional analysis during the temporal progression of fin regeneration. (**A**) Temporal co-expression network of all TFs during fin regeneration. (**B**) Heatmap of Z-score (mean-normalized expression level) for each subnetwork at all time points. (**C**) Temporal co-expression network of the candidate gene set. (**D**) Expression trend analysis of candidate genes in each subnetwork. The colors reflect the membership values, with darker shades representing higher membership. (**E**) Functional transitions of enriched pathways/terms for the candidate gene set during regeneration progression. (**F**) Temporal co-expression network of hub genes. (**G**) Line plot of expression trends for hub genes.

To investigate the temporal co-expression relationships of genes related to fin regeneration, TO-GCN analysis was performed. First, a GCN was constructed for all transcription factors (TFs), and then time ordering was performed based on seed genes to obtain seven regeneration-specific hierarchical networks (L1–L7) (Figure 4A). As shown by the red squares along the diagonal in the heatmap of Z score (average normalized RPKM) (Figure 4B), the hierarchical order of these sub-networks was consistent with the actual time sequence of regenerating samples. To further clarify the temporal progression, the seven subnetworks were aligned with specific regeneration stages, noting that multiple subnetworks may be active at a given time point. Specifically, at 0 dpa (immediate amputation), subnetworks L1–L3 are involved in early wound healing and initial signaling. At 1 dpa (early inflammation and blastema induction), L1–L5 contribute to the early regenerative response. At 3–4 dpa (active blastema formation and proliferation), L5–L7 become predominant, representing active differentiation and outgrowth. By 7 dpa (late tissue remodeling and functional maturation), L6–L7 are the main subnetworks.

The candidate gene set containing 82 genes screened by the 6 high-performance machine learning models was the focus of this study, and its temporal co-expression network is shown in Figure 4C. The expression trends of candidate genes in each sub-network showed significant dynamic changes with the regeneration process (Figure 4D), which were highly consistent with the temporal characteristics of the L2–L7 regeneration-specific hierarchical networks: genes in the sub-network corresponding to the L2 stage took the lead in initiating upregulation, triggering the molecular response after injury; candidate genes in each sub-network during the L3–L6 stages showed high expression synchronization, characterized by coordinated high expression, and with the progression of hierarchical levels, the time of expression peak occurrence gradually shifted backward; the expression levels of genes in the relevant sub-networks during the L7 stage gradually declined, while a small number of genes maintained high expression levels in the middle and late stages.

To complement these temporal expression trends with functional insights, we further analyzed how the biological roles of the candidate gene set shift across regeneration stages. Figure 4E illustrates this functional shift, showing that the enriched KEGG pathways and GO terms exhibit distinct stage-specific patterns, revealing a clear functional transition during regeneration progression. In the early stages (L2–L3), functions related to cytoskeleton remodeling and focal adhesion were predominantly enriched, reflecting the initiation of cell migration and proliferation. During the mid-stages (L4–L5), terms associated with extracellular matrix organization and ECM–receptor interaction became prominent, indicating active tissue remodeling and blastema growth. In the late stages (L6–L7), pathways related to motor proteins and hyaluronan metabolism were highly represented, suggesting a role in tissue maturation and structural stabilization. This functional transition, together with the expression trends observed in Figure 4D, demonstrates that zebrafish fin regeneration is governed not only by dynamic changes in gene expression levels but also by a systematic shift in the underlying biological functions across different temporal stages.

Hub genes and TFs were extracted separately from the co-temporal network of the candidate gene set to construct a temporal co-expression network. As shown in Figure 4F, the network clearly displayed the co-expression association characteristics of hub genes and TFs in each regeneration-specific hierarchical stage (L2–L7). The two formed specific co-expression connection patterns in each temporal stage, and the connection characteristics were consistent with the hierarchical progression trend of L2–L7.

Figure 4G shows the expression level change characteristics of 13 genes (including the above hub genes and TFs) at different actual dpa time points during fin regeneration, intuitively presenting the differences in expression levels of each gene in different actual time stages of regeneration. The expression levels of each gene showed different changing trends with the progression of dpa.

### 3.5. qPCR Validation of Temporal Expression Characteristics of Hub Genes and Transcription Factors

**Figure 5:**
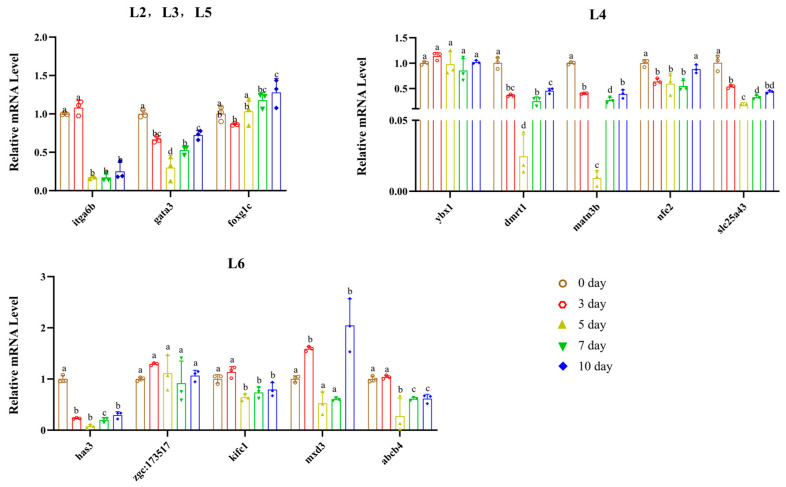
qPCR validation of the temporal expression patterns of core genes in different co-expression modules during zebrafish caudal fin regeneration. Error bars represent the standard error of the mean (SEM). Scatter points on each bar denote the raw data of individual biological replicates (n = 3). Different lowercase letters (a, b, c, ...) above the bars indicate significant differences among groups (one-way ANOVA followed by Tukey’s multiple comparison test, *p* < 0.05), and identical letters indicate no significant difference among groups.

qPCR-targeted validation results (Figure 5 and Figure S1) showed that the temporal expression patterns of core genes in different modules were highly consistent with the multi-omics transcriptomic analysis results, and supplemented the expression characteristics in the late regeneration stage (10 dpa).

In the L2, L3, and L5 modules, *itga6b* maintained high expression in the early regeneration stage (0–3 dpa) and significantly declined after 5 dpa; *gata3* showed stage-specific expression with a trend of first decreasing and then increasing; the expression level of *foxg1c* continuously increased with the regeneration process and reached its peak at 10 dpa. *ybx1*, *dmrt1*, *matn3b*, *nfe2*, and *slc25a43* in the L4 module were significantly activated in the early regeneration stage (0–3 dpa), downregulated in the middle stage (5 dpa), and partially recovered in the late stage (10 dpa). Genes such as *has3*, *kifc1*, and *mxd3* in the L6 module were significantly upregulated in the middle and late regeneration stages (3–7 dpa), among which *mxd3* reached the highest expression level at 10 dpa; *zgc:173517* and *abcb4* also showed the core characteristic of high expression in the middle and late stages. In this study, qPCR experiments successfully validated the temporal expression characteristics of core hub genes and transcription factors obtained by multi-omics screening at the transcriptional level, confirming the reliability of the multi-source transcriptomic integration analysis results.

### 3.6. Correlation Between Fin Regeneration and Immune Cells

**Figure 6:**
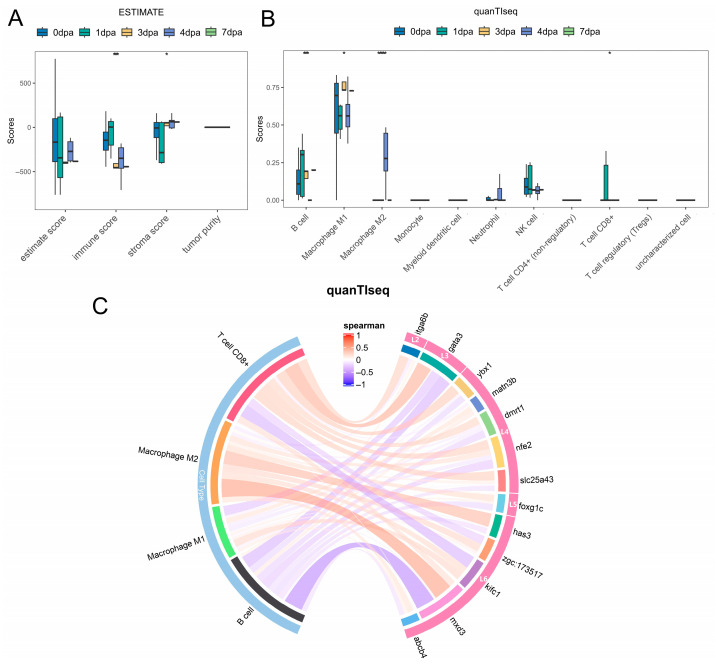
Results of immune infiltration analysis. (**A**,**B**) Scores and differential significance statistics of various immune cell types obtained by ESTIMATE and quanTIseq methods, respectively. Asterisks denote significance levels with * for *p* < 0.05, *** for *p* < 0.001, and **** for *p* < 0.0001. (**C**) Correlation analysis between hub genes, related TFs, and immune cell types with significant differences in infiltration levels.

To explore the correlation between zebrafish fin regeneration and immune cells, two types of immune cell infiltration analyses (ESTIMATE and quanTIseq) were performed. The results of the ESTIMATE method (Figure 6A) showed that the immune score exhibited a trend of first increasing and then decreasing. The quanTIseq method (Figure 6B, Supplementary Table S8) indicated that the infiltration levels of four types of cells (B cells, Macrophage M1, Macrophage M2, and T cell CD8+) changed significantly with the progression of fin regeneration.

We further calculated the correlation between hub genes, their related TFs, and these differential cell types. The results (Figure 6C) showed that with the progression of fin regeneration, these genes mainly exhibited promotion of T cell CD8+ in the early stage and inhibition in the late stage, promotion of Macrophage M2 cells in the late stage, and inhibition of B cells throughout the entire stage.

## 4. Discussion

Zebrafish fin regeneration serves as a classic model for vertebrate appendage regeneration. Deciphering its molecular regulatory mechanisms is one of the core scientific questions in the field of regenerative biology, holding significant reference value for understanding the conserved laws of tissue regeneration in aquatic vertebrates and providing practical insights for improving tissue repair strategies in farmed fish [10,23,53,54]. This study integrated multi-source bulk RNA-seq datasets related to zebrafish fin regeneration from the GEO public database, achieved standardized integration of multi-datasets through batch effect correction, and screened regeneration-related core gene sets by combining DE analysis with WGCNA. Furthermore, multiple machine learning algorithms were employed to accurately identify hub genes, and functional enrichment, temporal co-expression network construction, and immune infiltration analysis were performed to decipher the core characteristics of zebrafish fin regeneration from three dimensions: molecular function, dynamic regulation, and immune interaction. Additionally, qPCR technology was used for targeted validation of the temporal expression characteristics of core genes and supplementation of late-stage expression information. Notably, this study is a hypothesis-generating study, which refers to an exploratory analysis that uses large-scale omics data to identify candidate genes and generate new testable hypotheses rather than validate predefined mechanisms [55]. Its core objective is to identify hub candidate genes during zebrafish caudal fin regeneration through the integration of multi-source transcriptomes and machine learning screening, rather than directly validating the functional regulatory roles of these genes. However, the objective limitations based on technical means and analytical dimensions in this study indeed highlight specific directions for future research in this field.

### 4.1. Effectiveness of Multi-Source Data Integration and Core Gene Set Screening

Integrative analysis of multi-source public datasets is an effective approach to decipher complex biological processes, and the elimination of batch effects is a prerequisite for ensuring data reliability [24]. In this study, the ComBat method from the sva package was used for batch effect correction of three GEO datasets. Results of t-SNE dimensionality reduction clustering showed that batch differences among samples were significantly eliminated after correction, indicating the effectiveness of data standardization and laying a solid foundation for subsequent analyses. DE analysis identified 9394 significantly upregulated genes in zebrafish fins after injury, suggesting that the regeneration process involves the activation of a large set of genes and complex molecular regulatory responses. WGCNA constructed a gene co-expression network conforming to the scale-free network distribution by selecting the optimal soft threshold (power = 24), and identified the MEturquoise module with the highest correlation with the regeneration phenotype. Intersecting upregulated differential genes with genes in the gene module yielded 9203 core gene sets. The core advantage of this analytical strategy lies in balancing the individual expression characteristics of genes and the co-expression patterns among genes, overcoming the limitation that single DE analysis cannot reveal gene regulatory networks, and avoiding the inclusion of redundant genes without expression differences in single WGCNA. This effectively narrows down the range of candidate genes and provides a solid basis for the accurate screening of regeneration-related genes in subsequent studies. Although the correlation coefficients between the MEturquoise module and regeneration phenotype were relatively low (0.11 and 0.025), the genes in this module showed highly consistent expression patterns across all samples and were significantly enriched in key regeneration-related biological pathways including extracellular matrix organization and focal adhesion, which supports its important biological relevance in fin regeneration.

However, from the perspective of data resolution, this study was based on bulk RNA-seq data, which only reflects the average gene expression level of the entire sample and cannot resolve the heterogeneity of gene expression at the single-cell level. It is difficult to distinguish the gene expression patterns of different cell types (such as blastema cells, epithelial cells, and immune cells) during fin regeneration, nor can it clarify the cell-specific expression characteristics of the core gene set. As a result, the genes screened in this study only reflect the overall molecular changes during regeneration, failing to accurately locate the cellular targets where genes exert their functions. Meanwhile, during data collection, this study only included published bulk RNA-seq datasets, and the sample size was somewhat limited (Supplementary Table S1). The limited sample size may still affect the statistical power of some analyses. In addition, this study only included published bulk RNA-seq datasets, without integrating higher-resolution transcriptomic data such as single-cell RNA-seq and spatial transcriptome, nor combining multi-omics data including epigenome and proteome. This makes it difficult to decipher the molecular mechanisms of fin regeneration from the perspective of multi-level regulation (transcription, epigenetics, protein, etc.), limiting the molecular regulatory analysis of this study to the transcriptional level and failing to construct a multi-dimensional regulatory network.

### 4.2. Accurate Identification of Regeneration-Related Hub Genes and Their Functional Characteristics by Machine Learning Algorithms

This study is among the first to apply 13 classic machine learning algorithms for screening key genes related to zebrafish fin regeneration. Model performance evaluation showed that all algorithms had AUC values greater than 0.7, among which AdaBoost, XGBoost, Lasso, neural network, PLS, and Ridge exhibited the best performance with AUC values exceeding 0.9. The prediction accuracy of AdaBoost and XGBoost reached 100%, indicating that these models have high robustness and reliability, and are suitable for feature screening of regeneration-related genes. Integrating the genes identified by the 6 high-performance algorithms resulted in 82 candidate genes, and 10 hub genes (*trpc1*, *sst1.1*, *kifc1*, *has3*, *cyp1b1*, *matn3b*, *slc25a43*, *abcb4*, *sim1a* and *itga6b*) were further screened out, including ECM remodeling (e.g., *has3*, *matn3b*), cell adhesion (e.g., *itga6b*), and proliferation and metabolism (e.g., *ybx1*, *slc25a43*). With higher connectivity in the co-expression network, these genes constitute the core functional module during the middle and late stages of caudal fin regeneration. Among them, *has3* is induced following caudal fin amputation and maintains high expression in the wound epithelium; its catalysis of hyaluronic acid synthesis is essential for blastema formation and cell proliferation, and inhibition of *has3* activity leads to significant regeneration delay [56]. *ybx1* promotes progenitor cell proliferation and differentiation by regulating *atoh1a* expression, and its mutants exhibit regeneration-specific defects with approximately 20% delay in the initiation of hair cell regeneration [57]. The experimentally validated roles of these two genes are consistent with their identification as hub genes in our network analysis, confirming the reliability of our analytical strategy and providing a predictive basis for the regeneration-related functions of other candidate genes.

Notably, although ROC analysis indicates that these hub genes possess good discriminative power for regeneration phenotypes, and the constructed machine learning model shows favorable robustness within the integrated dataset, external or independent dataset validation was not performed in this study. Their generality should still be interpreted with caution. The model’s performance relies on the experimental design of the current dataset, and further validation is required for future applications to other injury types or regeneration models.

Functional enrichment analysis revealed that the candidate gene set was mainly enriched in KEGG pathways such as cytoskeleton in muscle cells, focal adhesion, and ECM–receptor interaction, as well as GO terms including hyaluronan metabolic process, extracellular matrix organization, and extracellular matrix structural constituent. These pathways and biological processes are all important processes associated with tissue regeneration. The remodeling of the extracellular matrix (ECM) is the basis for the regeneration of damaged tissues, which participates in the spatiotemporal regulation of the regeneration process by regulating cell adhesion, proliferation, migration, and differentiation [23]; focal adhesions, as important structures connecting cells and the ECM, are involved in the dynamic changes in the cytoskeleton and signal transduction, and play a crucial regulatory role in cell behavior during regeneration; hyaluronan metabolism participates in the synthesis and degradation of the ECM, affecting the formation of the regenerative microenvironment. As members of these relevant pathways, the high expression of the 10 putative hub genes suggests that they may potentially participate in the initiation and progression of zebrafish fin regeneration by regulating cytoskeleton remodeling, ECM organization, cell adhesion, and other processes, providing key targets for subsequent deciphering of regenerative molecular regulatory mechanisms.

### 4.3. Temporal Gene Regulatory Network Characteristics of Zebrafish Fin Regeneration

Tissue regeneration is a highly dynamic temporal process, and gene expression patterns exhibit significant stage-specific characteristics [58,59,60]. Deciphering the temporal gene regulatory network is crucial for understanding the molecular mechanisms of regeneration. This study constructed a temporal gene co-expression network of zebrafish fin regeneration using the TO-GCN method. By constructing a GCN for transcription factors (TFs) and performing time ordering, seven regeneration-specific hierarchical networks (L1–L7) were obtained, and the hierarchical order of the sub-networks was highly consistent with the actual time sequence of regenerating samples. This indicates that the temporal network can effectively reflect the dynamic changes in gene expression during regeneration. Analysis of TO-GCN and expression trends of the candidate gene set revealed two complementary layers of dynamic regulation during zebrafish fin regeneration. First, the expression levels of candidate genes exhibited clear stage-specific characteristics (Figure 4D), with distinct activation patterns across the L2 to L7 hierarchical stages, reflecting the finely tuned transcriptional dynamics underlying regeneration progression. Second, and more importantly, the biological functions performed by these genes underwent a systematic shift as regeneration advanced (Figure 4E). Early stages (L2–L3) were dominated by functions related to cytoskeleton remodeling and focal adhesion, supporting the initiation of cell migration and proliferation. Mid-stages (L4–L5) shifted toward extracellular matrix organization and ECM–receptor interaction, indicating active tissue remodeling and blastema growth. Late stages (L6–L7) were characterized by pathways associated with motor proteins and hyaluronan metabolism, pointing to roles in tissue maturation and structural stabilization. Together, these findings demonstrate that zebrafish fin regeneration is governed not only by temporal changes in gene expression levels, but also by a coordinated transition in the underlying biological functions across different regenerative stages.

qPCR-targeted validation experiments verified the temporal expression characteristics of core hub genes and transcription factors in modules L2, L3, L4, L5, and L6. The results were highly consistent with multi-omics temporal analysis, not only confirming the reliability of high-throughput analysis results but also extending the detection time point to 10 dpa, supplementing gene expression information in the late regeneration stage, and improving the full-cycle temporal regulatory map of core genes. For example, multi-omics analysis revealed that *foxg1c* showed an upward trend in the middle and late regeneration stages, while qPCR experiments further found that its expression peaked at 10 dpa, clarifying the role of this gene in tissue maturation and functional reconstruction in the late regeneration stage; the mid-stage downregulation and partial late-stage recovery of genes in the L4 module were also confirmed by qPCR experiments. In addition, the temporal co-expression relationships between hub genes and related TFs revealed the role of TFs in regulating the expression of hub genes.

### 4.4. Stage-Specific Interaction Between Fin Regeneration and the Immune Microenvironment

The immune microenvironment modulates tissue regeneration by regulating inflammatory responses and tissue repair [61,62,63,64]. Here, we conducted exploratory immune infiltration analysis via ESTIMATE and quanTIseq, and noted critically that both algorithms are validated for human/mammalian transcriptomic data, while zebrafish have a teleost-specific immune system with distinct cell markers and expression profiles [65,66]. Therefore, all results are computational predictions rather than experimentally verified data, and no definitive mechanistic conclusions on immune-gene crosstalk are drawn.

Exploratory results showed that the immune score during fin regeneration rose first and then declined, consistent with prior zebrafish studies [67,68]; quanTIseq identified B cells, M1/M2 macrophages and CD8+ T cells as having dynamically changing predicted infiltration levels. Correlation analysis revealed potential stage-specific association trends between hub genes/TFs and these cell types, which provide preliminary exploratory clues for immune-regeneration crosstalk in zebrafish.

This analysis has notable limitations: the predicted results lack experimental validation (e.g., flow cytometry, immunofluorescence), and the cross-species deconvolution methods restrict the interpretability of results. Further studies should adopt zebrafish/teleost-specific immune markers and validated deconvolution approaches, and combine in vivo/in vitro experiments to verify the actual interaction between hub genes and immune cells during fin regeneration.

Future studies can combine single-cell RNA-seq and spatial transcriptome technologies to decipher the cell-specific expression patterns and spatial localization characteristics of core hub genes, thereby clarifying the cellular targets through which these genes exert their regulatory functions during fin regeneration. Additionally, functional validation of hub genes via gene editing, in vivo imaging, and cell co-culture experiments will help decipher their upstream regulatory factors and downstream signaling pathways, as well as the specific molecular mechanisms underlying their interaction with immune cells. Integrating multi-omics data (e.g., epigenomics, proteomics, and metabolomics) will further construct a comprehensive multi-dimensional temporal regulatory network of zebrafish fin regeneration, overcoming the limitations of single-layer transcriptomic analysis. Furthermore, extending this analytical framework to other aquatic vertebrate species will enable exploration of the evolutionary conservation of regenerative mechanisms, providing a broader theoretical basis for comparative regenerative biology research. Ultimately, this will deepen our understanding of the intrinsic molecular and immune regulatory networks governing vertebrate tissue regeneration, laying a solid foundation for subsequent translational research in animal biology.

## 5. Conclusions

This study systematically identified putative hub genes associated with zebrafish fin regeneration and characterized the molecular pathways they participate in, their temporal regulatory characteristics, and interaction patterns with the immune microenvironment via multi-source data integration, combined screening strategies, multi-dimensional bioinformatics analysis, and qPCR validation. These findings provide potential gene targets and experimental evidence for vertebrate appendage regeneration research and help to further investigate the regenerative mechanisms of aquatic vertebrates, while offering a referential analytical paradigm for regenerative biology studies utilizing animal models. Despite these valuable insights, this study has inherent limitations related to data resolution and analytical depth that warrant further exploration in future work. Furthermore, the hub genes and regulatory pathways identified herein serve as potential targets and a theoretical foundation for investigating tissue injury repair in aquatic animals (e.g., farmed cyprinids), while facilitating translational research on vertebrate regenerative biology. This work bridges basic animal science with practical applications relevant to aquaculture and animal welfare, highlighting its value for both fundamental research and applied animal sciences.

## Figures and Tables

**Table 1:** Specific primers for verified genes were used for qRT-PCR.

Gene Name	Forward Primer	Reverse Primer
*itga6b*	TGTTTGTGTACAGGTCGCGT	ACCCTGATGGTGTATGCCAC
*gata3*	CGCCACCTCACAGTAGTCAG	GGACAGCGAGGAGGAAGAAG
*foxg1c*	GGAACAGATGTGCCGGAGAA	ACGAAGCACTTGTTGAGGCT
*ybx1*	GGCTTCCGACCAAGGGGT	GGCTGATTTGTCGGCTGATG
*dmrt1*	CCAACCAACCTAGGCAGTCG	CGGCCATTTCCACTAGGCAT
*matn3b*	TGACAGATGGAAGACCGCAA	CAAGCGTCGAAACCACAGAG
*nfe2*	CTGGTTTGTCGCTTGGTTCG	ACCGGACTGTAAGTGTGCAG
*slc25a4*	GCTGATATCGGCAAAGGTGC	GGGATCCGGCAACATACCTTTA
*has3*	TGTGTGGTGTGGAAAGGGAAT	GTGTCTGAATCACATACCTGCAT
*zgc:173517*	ACTCGTGTTGAAGATGAGAAGA	GTGTCCTCCATCAGGTCTGTTT
*kifc1*	TTCGCAACATACAGACTCAGC	CGGCCGAACCCTACAAAACA
*mxd3*	GCAACATCCAAGTGCTTCTGC	ATGTCTTAACTGAGCTCTCCTGTG
*abcb4*	AGCTCTAGATAAGGTGAGGCT	CATGTCTGAACAGTGGGTTTCT

Primer sequences are presented in the 5′→3′ direction.

## Data Availability

The data presented in this study were derived from public domain resources in the NCBI Gene Expression Omnibus (GEO) database at https://www.ncbi.nlm.nih.gov/geo/ (accessed on 2 December 2025) under the accession numbers GSE126701, GSE146960 and GSE160909. No new sequencing data were generated in this study.

## Supplementary Materials

The following supporting information can be downloaded at: https://www.mdpi.com/article/10.3390/genes17050503/s1, Figure S1: Line plot of qPCR-validated temporal expression patterns of core genes in different co-expression modules during zebrafish caudal fin regeneration; Tables S1–S8: Sample information and results of bioinformatics analysis.
